# Energy-Efficient and Adversarially Resilient Underwater Object Detection via Adaptive Vision Transformers

**DOI:** 10.3390/s25226948

**Published:** 2025-11-13

**Authors:** Leqi Li, Gengpei Zhang, Yongqian Zhou

**Affiliations:** The School of Electronic Information and Electrical Engineering, East Campus, Yangtze University, Jingzhou 434100, China; lileqi2023@outlook.com (L.L.); judgebill@126.com (G.Z.)

**Keywords:** underwater object detection, adaptive vision transformer, image enhancement, energy efficiency optimization, adversarial robustness, security defense

## Abstract

Underwater object detection is critical for marine resource utilization, ecological monitoring, and maritime security, yet it remains constrained by optical degradation, high energy consumption, and vulnerability to adversarial perturbations. To address these challenges, this study proposes an Adaptive Vision Transformer (A-ViT)-based detection framework. At the hardware level, a systematic power-modeling and endurance-estimation scheme ensures feasibility across shallow- and deep-water missions. Through the super-resolution reconstruction based on the Hybrid Attention Transformer (HAT) and the staged enhancement with the Deep Initialization and Deep Inception and Channel-wise Attention Module (DICAM), the image quality was significantly improved. Specifically, the Peak Signal-to-Noise Ratio (PSNR) increased by 74.8%, and the Structural Similarity Index (SSIM) improved by 375.8%. Furthermore, the Underwater Image Quality Measure (UIQM) rose from 3.00 to 3.85, while the Underwater Color Image Quality Evaluation (UCIQE) increased from 0.550 to 0.673, demonstrating substantial enhancement in both visual fidelity and color consistency. Detection accuracy is further enhanced by an improved YOLOv11-Coordinate Attention–High-order Spatial Feature Pyramid Network (YOLOv11-CA_HSFPN), which attains a mean Average Precision at Intersection over Union 0.5 (mAP@0.5) of 56.2%, exceeding the baseline YOLOv11 by 1.5 percentage points while maintaining 10.5 ms latency. The proposed A-ViT + ROI reduces inference latency by 27.3% and memory usage by 74.6% when integrated with YOLOv11-CA_HSFPN and achieves up to 48.9% latency reduction and 80.0% VRAM savings in other detectors. An additional Image-stage Attack QuickCheck (IAQ) defense module reduces adversarial-attack-induced latency growth by 33–40%, effectively preventing computational overload.

## 1. Introduction

With the growing strategic demand for marine resource exploitation, ecological environment protection, and underwater military defense, underwater object detection has become an increasingly critical component of intelligent ocean perception. Its applications span multiple domains, including marine biodiversity monitoring, safety inspection of subsea pipelines and facilities, autonomous navigation of unmanned underwater vehicles (UUVs), and underwater security operations.

However, a fundamental and highly topical challenge in underwater target detection is the issue of energy consumption and system security: the constrained power budgets of underwater platforms, the harsh and variable optical conditions, and the potential for adversarial or fault-driven failures demand detection systems that are not only accurate but also energy-efficient, robust, safe, and reliable. Prior work in marine sensing and underwater image systems has highlighted that energy-aware design is essential for long-term autonomous operation and mission assurance in subsea environments [1,2].

Image enhancement is an indispensable preprocessing step to ensure subsequent detector performance in underwater object detection. In general computer vision tasks, conventional methods such as histogram equalization, contrast stretching, adaptive histogram equalization, and color normalization are often applied. While these techniques can temporarily improve visual quality, they fail to address the non-uniform degradations, color shifts, and detail losses commonly present in underwater imagery. To overcome the limitations of traditional approaches in complex underwater environments, recent studies have increasingly adopted hybrid strategies that combine physical modeling with deep learning. These methods go beyond simple contrast adjustments by incorporating optical priors, generative adversarial networks (GANs), and Transformer architectures to model degradation more effectively. For instance, the DICAM network deeply integrates channel compensation with attention mechanisms, enabling natural color restoration and detail enhancement while maintaining physical interpretability [3]; FunIEGAN leverages an adversarial framework for real-time enhancement through end-to-end training, significantly improving the perceptual quality of underwater targets [4]; Rep-UWnet employs lightweight convolutions and residual designs to achieve strong enhancement performance with minimal computational cost, making it suitable for embedded deployment [5,6]. More recent approaches such as TOPAL and UDAformer represent emerging trends, utilizing adversarial-perception optimization and the global modeling capacity of Transformers, respectively, to address non-uniform degradation while preserving structural consistency [7,8,9]. Collectively, these advances mark a shift from “experience-driven” enhancement to “physics- and data-driven” paradigms, laying a solid foundation for high-quality underwater perception and detection.

Beyond image enhancement, detection models themselves play a decisive role in underwater recognition tasks. Traditional two-stage detectors such as Faster R-CNN [10] offer high accuracy in general vision tasks but are difficult to deploy on underwater platforms with real-time constraints due to their complex region proposal and feature alignment steps [11,12,13]. In contrast, single-stage detectors such as YOLO and SSD, with their end-to-end structures and faster inference speeds, have become mainstream in underwater detection. However, its performance degrades significantly in underwater environments due to challenges such as light attenuation, scattering noise, and small target sizes. Specifically, two major limitations hinder its effectiveness for underwater object detection: (i) insufficient spatial directionality, which results in blurred target boundaries, and (ii) difficulty in distinguishing small or low-contrast targets from cluttered backgrounds, leading to missed and false detections. Recent works have proposed various improvements: for example, the SWIPENET framework introduces multi-scale attention to enhance small-object detection and robustness to noise [14]; U-DECN integrates deformable convolutions and denoising training into the DETR framework, boosting deployment efficiency [15]; NAS-DETR combines neural architecture search with FPN and Transformer decoders, achieving promising results on both sonar and optical underwater imagery [16]; PE-Transformer and AGS-YOLO demonstrate the potential of Transformers and multi-scale fusion for underwater detection [17,18]; meanwhile, YOLOv5 variants incorporating semantic modules and attention-based detection heads further improve small-object detection [19]. These studies highlight that although progress has been made, existing detection models still face trade-offs between accuracy and efficiency under complex degraded conditions [20].

The introduction of Transformer architectures offers new opportunities for underwater detection. Standard Vision Transformers (ViTs) possess strong global modeling capability, enabling them to capture long-range dependencies and handle complex background-target interactions [21]. However, their high computational cost and large memory footprint severely constrain deployment on resource-limited platforms such as UUVs [22,23]. Existing surveys have also pointed out that while Transformers show promise in underwater detection, systematic studies on energy optimization and adversarial adaptation remain lacking [24,25].

Recent research has explored multiple solutions: ViT-ClarityNet integrates CNNs and ViTs to enhance low-quality image representation [26]; FLSSNet leverages a CNN–Transformer–Mamba hybrid structure to improve long-range dependency modeling and noise suppression [27]; the RVT series emphasizes position-aware attention scaling and patch-wise augmentation to enhance robustness [28]; AdvI-token ViT introduces adversarial indicator tokens to detect perturbations during inference [29]. These developments form the methodological basis of our Adaptive Vision Transformer (A-ViT), whose dynamic token pruning and early-exit strategies aim to reduce energy consumption while maintaining accuracy and enhancing robustness in degraded and adversarial scenarios.

In adversarial underwater environments, security defense is equally critical: the inherently low quality of underwater images makes adversarial perturbations difficult to detect visually, thereby creating opportunities for stealthy attacks. In underwater settings, adversarial patch attacks can even be disguised as coral textures or sand patches, making them stealthier and more dangerous [30]. Previous research has shown that unmodified Vision Transformer architectures are especially susceptible to adversarial manipulations, with unstable internal representations even under minimally perturbed inputs [28,31,32]. Related reviews also emphasize that practical deployment must account for distribution shifts, noise interference, and intentional attacks, advocating for adversarially robust frameworks [12,33]. For example, AdvI-token ViT introduces robustness-discriminating tokens at the input layer to improve adversarial detection [29].

Therefore, this study addresses not only energy efficiency and detection accuracy but also proposes defense strategies against novel threats such as energy-constrained attacks, ensuring the security and usability of detection frameworks. However, current defense mechanisms remain insufficient: most focus on natural or general image tasks, with limited consideration of underwater-specific degradations and low-quality conditions. Additionally, existing defenses are typically confined to single levels (e.g., adversarial training or input correction), lacking integrated, multi-level strategies spanning image enhancement, detection optimization, and Transformer-level defense. This gap is particularly evident on resource-limited underwater platforms, where models may fail to deploy despite promising laboratory results due to excessive energy consumption or adversarial vulnerabilities [34].

In summary, despite substantial progress in image enhancement and detection optimization, current underwater object detection systems still suffer from severe trade-offs between energy consumption and detection stability. High-performance networks often achieve accuracy at the cost of excessive computational demand, making real-time deployment on power-constrained underwater platforms infeasible. Therefore, developing an underwater detection framework that can significantly reduce energy consumption without compromising detection stability has become both a scientific necessity and an engineering priority.

To guide the reader, the remainder of this paper is structured as follows. Section 2 develops the theoretical foundations: Section 2.1 formalizes the underwater imaging hardware design; Section 2.2 presents the image processing module, detailing Section 2.2.1 HAT-based super-resolution and Section 2.2.2 staged underwater enhancement; Section 2.3 introduces the improved YOLOv11-CA_HSFPN detector; Section 2.4 describes the Adaptive Vision Transformer (A-ViT) for adversarial underwater scenarios with ROI-aware modeling; and Section 2.5 analyzes energy-constrained adversarial attacks and the resulting vulnerabilities of A-ViT. Section 3 reports experiments: Section 3.1 quantifies hardware design and material choices; Section 3.2 evaluates the image processing module (with Section 3.2.1 super-resolution and Section 3.2.2 multi-stage enhancement); Section 3.3 visualizes YOLOv11-CA_HSFPN detection; Section 3.4 and Section 3.5 provide dynamic visualizations of fish-school detection with A-ViT and verify adversarial vulnerabilities; and Section 3.6 assesses the IAQ image-stage quick defense. Section 4 offers discussion and ablations: Section 4.1 on image processing modules, Section 4.2 on YOLOv11-CA_HSFPN, Section 4.3 comparative analysis with representative underwater detectors, Section 4.4 examination of the A-ViT+ROI dynamic inference mechanism, and Section 4.5 the energy–efficiency vulnerability of A-ViT+ROI under attack. Section 5 concludes the study and outlines future research directions.

## 2. Theoretical Analysis

**Figure 1:**
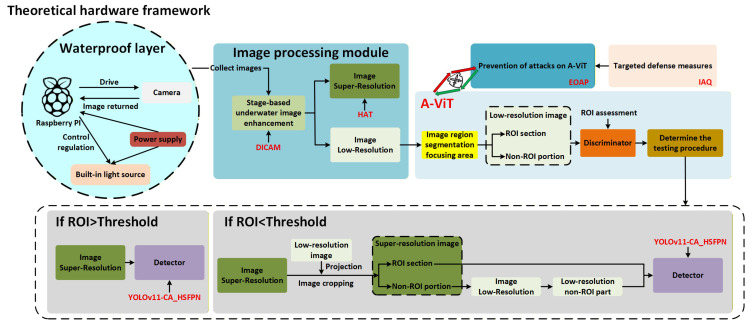
System architecture of underwater object detection.

As shown in Figure 1. This paper proposes an Adaptive Vision Transformer (A-ViT)-based underwater detection framework that achieves breakthroughs across image enhancement, lightweight detection, energy-efficient modeling, and multi-level adversarial defense. The main contributions of this work are as follows:

1. Underwater imaging hardware framework: A theoretical analysis system is established covering power modeling, endurance estimation, and thermal constraints. Combined with material selection and empirical data, a feasible solution is proposed for shallow-, mid-, and deep-water mission modes.
2. Image processing module: HAT-based super-resolution and DICAM staged enhancement are integrated for clarity restoration, color correction, and contrast enhancement. Experiments and ablation studies confirm that this module substantially improves detection accuracy.
3. Adaptive Vision Transformer (A-ViT): Dynamic token pruning and early-exit strategies enable on-demand computation, significantly reducing latency and GPU memory usage. Together with fallback and key-region preservation mechanisms, A-ViT enhances robustness under adversarial perturbations.
4. Improved detector architecture (YOLOv11-CA_HSFPN): Coordinate attention and a high-order spatial feature pyramid are incorporated into the neck, boosting detection of small and blurred objects while providing accuracy and robustness gains with minimal additional computational cost.
5. Adversarial Vulnerability and Theoretical Defense: The inherent vulnerability of A-ViT to energy-oriented adversarial perturbations (EOAPs) is identified, and a theoretical multi-level defense is formulated. By integrating the lightweight Image-stage Attack QuickCheck (IAQ) and robustness-aware halting regularization, the framework ensures stable, energy-bounded inference and enhanced resilience under adversarial conditions.

Collectively, this work establishes a closed-loop design that integrates hardware feasibility, image preprocessing, energy-efficient Transformer modeling, and optimized detector architecture, thereby providing a systematic solution for building efficient, robust, and secure underwater intelligent perception systems.

### 2.1. Underwater Imaging Hardware Design

**Figure 2:**
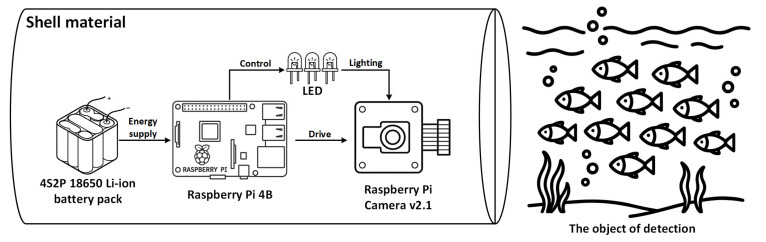
Theoretical Framework of the Hardware System.

In this section, a theoretical framework for power modeling, endurance estimation, and material selection is established, as illustrated in Figure 2.

The system mainly consists of the following components:

1. Main control platform: Embedded processing unit, with power consumption denoted as $P_{RP}$.
2. Imaging device: Image sensor or camera module, with power consumption denoted as $P_{\mathrm{cam}\;}$.
3. Illumination module: Light source unit with power consumption denoted as $P_{LED}$ and duty cycle $D_{LED}$, where $D_{LED}$ increases with operating depth.
4. Power supply system: Battery pack with nominal voltage $V_{b}$, capacity $C_{b}$, and total energy:

(1) $$E_{b}=V_{b}\times C_{b}$$

The instantaneous system power consumption is determined by the power of each module and its duty cycles:

(2) $$P_{\mathrm{tot}\;}=P_{RP}+P_{\mathrm{cam}\;}+D_{LED}P_{LED}+D_{\inf\;}\Delta P_{alg}$$

where $D_{\inf\;}$ is the duty cycle of algorithm execution and $\Delta P_{alg}$ represents the incremental power induced by algorithmic operations.

Accordingly, the average system power can be expressed as:

(3) $$\bar{P}=P_{RP}+P_{cam}+D_{LED}P_{LED}+D_{inf}\Delta P_{alg}$$

Considering power efficiency and the usable energy coefficient, the theoretical endurance time of the battery is:

(4) $$T_{\mathrm{run}\;}=\frac{\kappa E_{b}\eta }{\bar{P}}$$

where κ is the usable energy coefficient, ranging from 0.7 to 0.9, accounting for depth of discharge and temperature effects, and η is the power conversion efficiency, ranging from 0.85 to 0.95.

To further quantify system performance during the imaging process, a per-frame energy consumption index is introduced:

(5) $$E_{\mathrm{frame}\;}=\frac{\bar{P}}{F}$$

where F is the frame rate per second. $E_{\mathrm{frame}\;}$ represents the average energy required to capture a single image. For engineering comparison, this can be converted into energy per thousand frames:

(6) $$E_{1000}=\frac{1000\bar{P}}{3600\bar{F}}$$

with units of watt-hours per thousand frames, which can serve as a direct quantitative metric for comparing different systems and algorithms, measured in Wh per 1000 frames.

In addition, thermal constraints must be satisfied to avoid system overheating:

(7) $$A\geq \frac{\bar{P}}{h\Delta T_{max}}$$

where A is the effective surface area of the enclosure required for heat dissipation in square meters, h is the convective heat transfer coefficient in water, ranging from 200 to 800 $W\cdot m^{-2}\cdot K^{-1}$, and $\Delta T_{max}$ is the allowable steady-state temperature rise.

In summary, this section establishes a theoretical analysis framework for underwater imaging hardware, covering system composition, power modeling, endurance estimation, and thermal dissipation constraints. Through the derivation of Equations (1)–(7) and parametric modeling, the framework enables prediction of energy consumption and endurance across varying task duty cycles. Furthermore, material selection for the housing and optical window is analyzed in the experimental section, where thermal conductivity, structural strength, and environmental adaptability are quantitatively evaluated to provide a theoretical basis for engineering design.

### 2.2. Image Processing Module

#### 2.2.1. Image Super-Resolution

To improve underwater image quality, we introduce the Hybrid Attention Transformer for ImageRestoration (HAT) [35] in the image preprocessing module. HAT restores degraded textures and edges by leveraging hierarchical feature modeling and cross-channel attention. Unlike traditional convolution-based super-resolution methods, HAT combines the global modeling power of Transformers with the local feature extraction capability of convolutions. This makes it particularly effective at addressing the complex and non-uniform degradation found in underwater images.

**Figure 3:**
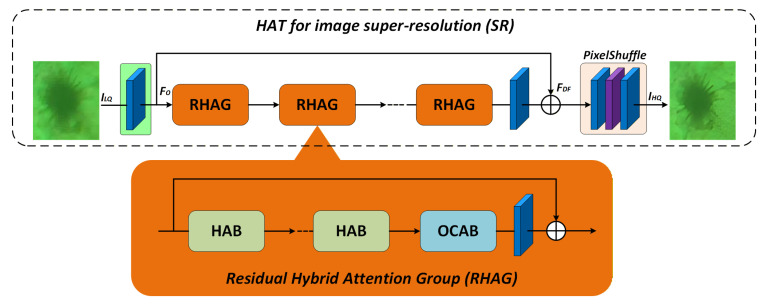
Network architecture of HAT for image super-resolution.

As illustrated in Figure 3, the HAT architecture consists of three main components: shallow feature extraction, deep feature construction via stacked residual attention groups, and final high-resolution reconstruction. Specifically, the input low-quality image $I_{LQ}$ is first mapped to shallow features $F_{0}$. These features are then processed through N stacked Residual Hybrid Attention Groups (RHAGs), followed by up-sampling using PixelShuffle, which outputs the high-resolution image $I_{HQ}$:

(8) $$I_{HQ}=\mathrm{PixelShuffle}\left({RHAG}^{N}\left(F_{0}\right)\right)$$

Within each RHAG, residual connections facilitate feature propagation, while attention mechanisms enhance cross-channel and cross-domain feature interactions. The formulation is expressed as:

(9) RHAG(x)=x+HATBlock(x)

The Hybrid Attention Block (HAB) serves as the core unit of RHAG. By integrating window-based multi-head self-attention (W-MSA) with local convolutions, HAB establishes joint modeling of long-range dependencies and local correlations. The self-attention operation is defined as:

(10) $$Attention(Q,K,V)=Softmax\left(\frac{QK^{T}}{\sqrt{d}}+B\right)V$$

where Q, K, and V denote the query, key, and value matrices, respectively; d is the feature dimension; and B is a learnable positional bias term, enabling enhanced positional encoding and sensitivity.

To alleviate vanishing gradient and convergence issues in deep residual training, HAT introduces LayerScale residual modulation at the output stage, formulated as:

(11) $$Output=x+\gamma_{1}\cdot Attention(x)+\gamma_{2}\cdot MLP(x)$$

where $\gamma_{1}$ and $\gamma_{2}\;$ are learnable scaling parameters that stabilize gradient propagation and improve adaptivity during early-stage training.

HAT incorporates the Overlapping Cross-Attention Block (OCAB), which aggregates overlapping window regions to improve boundary representation, enabling RHAG to achieve synergistic enhancement across multiple scales and improve sharpness and structural fidelity in the reconstructed images. Additionally, PixelShuffle is employed to upsample feature maps, converting low-resolution outputs into high-resolution images. This operation ensures high-quality reconstruction while maintaining computational efficiency, making it particularly suitable for energy-constrained applications such as underwater object detection.

In summary, the HAT framework effectively integrates hierarchical feature modeling with cross-channel attention to address the complex degradation typically found in underwater images. By combining the global modeling power of Transformers with the local feature extraction of convolutions, HAT ensures high-quality image restoration while maintaining computational efficiency, making it particularly suitable for energy-constrained underwater object detection tasks.

#### 2.2.2. Staged Underwater Image Enhancement

To address common issues in underwater images such as color imbalance, insufficient contrast, and blurred textures, this study adopts a staged enhancement strategy that integrates physics-based priors with deep learning, with the overall workflow as follows:

(1) Physics-based modeling of degradation.

We begin with a classical underwater degradation model to describe the formation process:

(12) I(x)=J(x)⋅t(x)+B⋅(1−t(x))

where I(x) is the observed image, J(x) is the latent clean image, t(x) denotes transmission, and B represents background light intensity.

To compensate for severe color cast caused by rapid attenuation of red light, a channel-wise compensation mechanism is introduced. The corrected image for channel c∈{R,G,B} is expressed as:

(13) $$J_{c}(x)=\frac{I_{c}(x)-B_{c}}{t(x)}+B_{c}$$

where $I_{c}(x)$ is the observed value in channel c, and $B_{c}$ is the corresponding background light. This formulation explicitly restores red-channel attenuation and re-balances three-channel intensity.

(2) Deep learning-based enhancement with DICAM.

**Figure 4:**
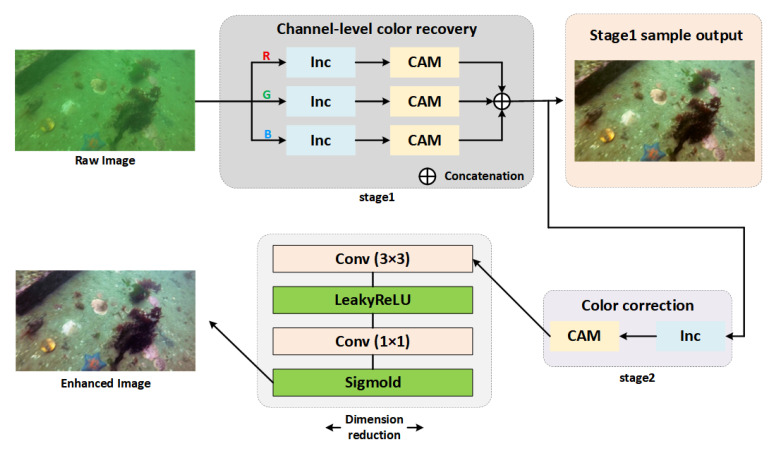
Network architecture of the DICAM for staged underwater image enhancement.

Building upon the physics-based correction, we introduce a Deep Image Correction and Adaptive Modeling (DICAM) network, as shown in Figure 4, to refine local structures and restore visual fidelity. The overall process can be formulated as:

(14) $$J^{*}(x)=F_{\theta }(J(x),t(x),B)$$

where $F_{\theta }$ represents a nonlinear mapping parameterized by deep network weights θ, which integrates physics-based priors with learned feature representations.

Stage 1: Channel-level recovery. The physics-corrected R/G/B channels are processed with channel attention modules (CAMs) to refine inter-channel correlations and suppress severe chromatic bias. Overlapping local information is aggregated to strengthen structural edges and reduce scattering noise.

Stage 2: Color correction and dimensional reduction. A compact module comprising convolution (3 × 3), LeakyReLU, and convolution (1 × 1) layers compresses redundant features, followed by a Sigmoid activation to produce final enhanced outputs.

In our staged strategy, the physics-based correction and DICAM-based enhancement work in a progressive loop: the physical model handles global degradation, while DICAM refines local structures and adaptive details. This approach ensures comprehensive restoration across various underwater conditions.

DICAM implements a hybrid approach of physics-guided and data-adapted fusion. In our staged strategy, the physics-based correction addresses global degradation, while DICAM refines local structures and adaptive details. The effectiveness of this method will be validated through subsequent experiments.

### 2.3. Improved Detector: YOLOv11-CA_HSFPN

**Figure 5:**
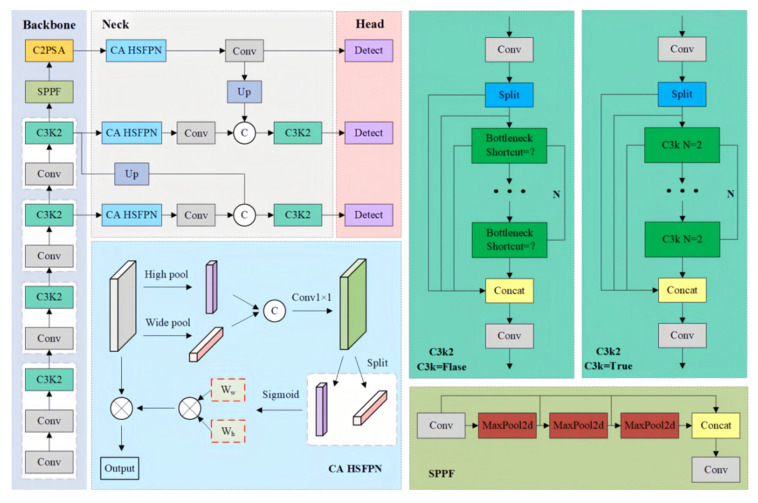
Network structure of the improved YOLOv11-CA_HSFPN.

This study proposes an improved architecture, YOLOv11-CA_HSFPN, shown in Figure 5. In this design, a Coordinate Attention–High-order Spatial Feature Pyramid Network (CA-HSFPN) module is incorporated into the neck to enhance spatial dependency modeling and improve small-target recognition.

We will analyze the Coordinate Attention (CA) and High-order Spatial Feature Pyramid Network (HSFPN) modules in the following content.

1. **Coordinate Attention (CA).**

The CA mechanism captures long-range dependencies while preserving precise positional information. Given an input feature $X\in R^{C\times H\times W}$, CA performs average pooling along height and width dimensions:

(15) $$x_{h}\left(c,i\right)=\frac{1}{W}\sum_{j=1}^{W}X\left(c,i,j\right),\;x_{w}\left(c,j\right)=\frac{1}{H}\sum_{i=1}^{H}X\left(c,i,j\right)$$

The pooled features are concatenated and compressed through a 1×1 convolution and nonlinear activation δ(⋅):

(16) $$f=\delta \left(W_{1}\left[x_{h},x_{w}\right]\right)$$

Directional weights are then generated via separate convolutions:

(17) $$a_{h}=\sigma \left(W_{h}f_{h}\right),\;a_{w}=\sigma \left(W_{w}f_{w}\right)$$

Finally, spatially aware feature refinement is achieved as:

(18) $$Y=X\cdot a_{h}\cdot a_{w}$$

This enables dynamic weighting of horizontal and vertical dependencies, strengthening elongated and boundary structures of underwater targets.

2. **High-order Spatial Feature Pyramid Network (HSFPN).**

On the basis of CA-enhanced features, HSFPN performs multi-level cross-scale fusion. The fused representation is expressed as:

(19) $$F_{n}^{\prime }=\sum_{i=1}^{L}U_{i}\left(CA\left(F_{b}^{\left(i\right)}\right)\right)$$

where $F_{b}^{\left(i\right)}$ denotes the backbone feature, CA(⋅) is coordinate attention, and $U_{i}(\cdot )$ represents up-sampling and concatenation operations. Compared with conventional FPNs, this approach preserves geometric sensitivity and is well suited to fine-grained distribution patterns of underwater targets.

The overall detection process of the improved architecture is:

(20) $$Y^{\prime }=\sigma \left(F_{\mathrm{Head}\;}\left(F_{\mathrm{CA}-\mathrm{HSFPN}\;}\left(F_{\mathrm{Backbone}\;}(X)\right)\right)\right)$$

In theory, compared with the original YOLOv11, the improved YOLOv11-CA_HSFPN offers the following benefits:

(1) Enhanced spatial directionality: Explicit modeling of long-range dependencies compensates for YOLOv11’s weakness in spatial feature capture.
(2) Improved small-target detection: CA-HSFPN facilitates discrimination of low-contrast small objects from noisy backgrounds, improving recall.
(3) Robustness reinforcement: Under challenging conditions such as low illumination and scattering, CA attention highlights structural cues, ensuring stable detection.
(4) Lightweight preservation: CA-HSFPN introduces only lightweight 1×1 convolutions and pooling operations, adding negligible computational cost.
(5) Underwater adaptability: Targets with orientation-dependent distributions, such as fish schools, achieve better representation under CA-HSFPN, aligning with underwater scene characteristics.

In summary, YOLOv11-CA_HSFPN achieves a balanced optimization between underwater detection performance and model efficiency. By enhancing feature representation while maintaining lightweight computation, it ensures accurate and robust detection under complex underwater conditions. This balance of precision and efficiency makes the model well suited for real-time applications in underwater monitoring and intelligent marine systems.

### 2.4. Adaptive Vision Transformer for Modeling in Adversarial Underwater Environments

An excellent underwater object detection system must achieve stable performance under conditions of insufficient illumination, color distortion, and suspended-particle scattering, while minimizing energy consumption to extend operational life.

**Figure 6:**
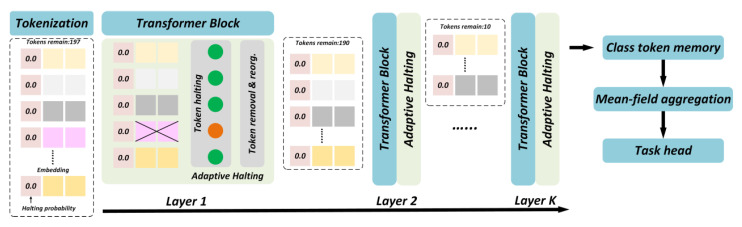
Illustration of the dynamic token pruning and early-exit mechanism in the proposed A-ViT.

Conventional Vision Transformers (ViTs) process all patch tokens uniformly, leading to high computational redundancy and excessive memory consumption, which restricts deployment on resource-limited underwater platforms. To address this challenge, we introduce an Adaptive Vision Transformer (A-ViT) that employs dynamic token selection and early-exit strategies to enable on-demand computation (Figure 6), thereby reducing computational overhead while maintaining robustness to perturbations.

The core concept of ViT is to assess the importance of input tokens adaptively and retain only the most discriminative subset for further computation. Suppose an input image is partitioned into N patches, each represented as a token vector $x_{i}\in R^{d}$. In a standard ViT, all tokens are passed through the Transformer encoder with complexity $𝒪(N^{2}\cdot d)$. For efficiency, A-ViT introduces a token selection function 𝒮(⋅), which ranks tokens based on joint attention entropy and feature compactness, selecting the most informative subset:

(21) $$𝒮=f(X)={Top}_{k}(\alpha \cdot H(x_{i})+(1-\alpha )\cdot \|A_{i}{\|}_{1})$$

where $X=\{x_{1},\ldots ,x_{N}\}$ is the token set, $H(x_{i})$ denotes information entropy, $\|A_{i}{\|}_{1}$ represents attention weight magnitude, and α is a balancing coefficient. The operator ${Top}_{k}(\cdot )$ selects the top-k tokens with the highest discriminability, discarding the rest to reduce computation.

A-ViT further incorporates early-exit branches within the Transformer layers. If the classification confidence of an intermediate output exceeds a threshold τ, the model directly outputs predictions without traversing deeper layers:

(22) $$\underset{c}{max}P\left(y=c|X^{\left(l\right)}\right)\geq \tau$$

where $X^{\left(l\right)}$ denotes the feature set at layer l. For simple samples, inference is terminated early to save energy; for complex or adversarially perturbed samples, deeper layers are automatically activated to extract fine-grained features, ensuring robust predictions.

To further strengthen stability under adversarial conditions, A-ViT introduces a robustness-aware importance index. Given a token $x_{i}$, adversarial noise δ, and perturbed token $x_{i}+\delta$, robustness importance is defined as:

(23) $$I\left(x_{i}\right)=E_{\delta ∼D}\left[{∥\begin{array}{c} f\left(x_{i}\right)-f\left(x_{i}+\delta \right) \end{array}∥}_{2}\right]$$

where D is the perturbation distribution. Tokens with lower sensitivity to perturbations are prioritized, improving accuracy and reliability in adversarial underwater environments.

For efficiency, A-ViT is combined with mixed-precision training and attention acceleration techniques (e.g., FlashAttention). To further optimize memory usage, we model effective computation area by separating foreground ROI tokens and background tokens.

1. Foreground ROI retention: Regions with high saliency are preserved at full resolution for YOLO-based detection, ensuring accurate boundary recall.
2. Background down-sampling: Non-salient regions are down-sampled (e.g., to 0.5× or 0.25× resolution) and processed only once, reducing redundancy.
3. Fallback mechanism: If ROI coverage drops below threshold, the model reverts to full-resolution processing to avoid missing potential targets.

The effective computational area can be expressed as:

(24) $$A_{\mathrm{eff}\;}=rA+\left(1-r\right)\cdot s^{2}A$$

where A is the total image area, rrr is the foreground ratio, and s is the background scaling factor.

For example, in a typical sparse-target scene with r=0.25 and s=0.5, the effective processed area reduces to ~44% of the original, with accuracy nearly unchanged, while peak memory and energy consumption are proportionally reduced.

In this framework, A-ViT acts not as a replacement detector but as a dynamic ROI regulator that prioritizes “focusing on salient objects while downplaying background clutter.” This design ensures that edge deployment can simultaneously meet memory and accuracy requirements, offering superior efficiency compared to full-image detection or purely visualization-driven approaches.

### 2.5. Energy-Constrained Adversarial Attacks and Vulnerability Analysis of A-ViT

**Figure 7:**
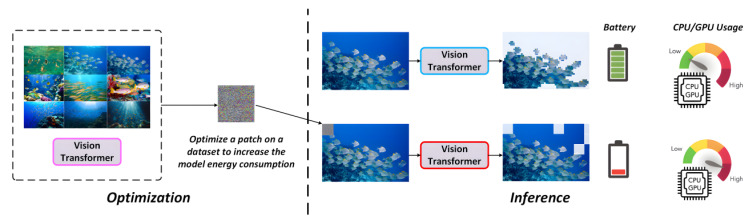
Illustration of EOAP-based adversarial disruption against A-ViT.

Recent studies have revealed that attackers can design Energy-Oriented Adversarial Patches (EOAPs) to exploit this mechanism. Specifically, EOAPs force the model to retain redundant or irrelevant tokens during inference, effectively degrading A-ViT into near full-scale computation. This significantly increases memory and computation cost, thereby eliminating the energy efficiency advantage of A-ViT. Figure 7 illustrates the EOAP perturbation mechanism.

The EOAP attack mechanism is interpreted through two components: (i) formulation of the halting mechanism and (ii) the EOAP adversarial objective. The former formalizes A-ViT’s early-exit process as optimizable metrics, thereby exposing the EOAP attack surface; the latter casts the attack as an optimization that perturbs this mechanism to suppress early exiting and increase computational cost, clarifying how the objective directly targets the underlying mechanism.

1. **Formulation of the Halting Mechanism**

For an input image partitioned into tokens $\{x_{i}{\}}_{i=1}^{N}$, the halting probability of token i at the first layer is denoted by $p_{i}^{\left(l\right)}$. A token terminates computation early once the accumulated halting probability exceeds a threshold:

(25) $$\sum_{l=1}^{L}p_{i}^{\left(l\right)}\geq 1-\epsilon$$

where $\tau_{i}$ is the halting layer index of token i. To prevent premature or delayed termination, A-ViT introduces a ponder loss during training:

(26) $$L_{\mathrm{ponder}\;}=\frac{1}{N}\sum_{i=1}^{N}{\left(\tau_{i}-\bar{\tau }\right)}^{2}$$

where $\bar{\tau }$ is the expected average halting depth. Additionally, to regularize the halting distribution toward a prior, a KL divergence penalty is employed:

(27) $$L_{KL}=D_{KL}\left(P_{\tau }∥P_{\mathrm{prior}}\right)$$

The final joint loss function is thus:

(28) $$L=L_{\det\;}+\alpha L_{\mathrm{ponder}\;}+\beta L_{KL}$$

where $L_{\det\;}$ is the detection task loss, and α, β are balancing coefficients to ensure efficient allocation of computation across easy and hard samples.

2. **EOAP Adversarial Objective**

Under adversarial environments, attackers explicitly manipulate the halting mechanism by applying perturbations δ. The optimization target is:

(29) $$\underset{\delta \in 𝒮}{min}E_{x∼D}\left[C\left(f\left(x+\delta \right)\right)\right]$$

where 𝒮 represents perturbation constraints such as amplitude bounds or masked regions, and C(⋅) denotes computational cost measured by the number of tokens retained or FLOPs. This objective enforces the retention of more tokens during inference, driving A-ViT toward near-full computation and thereby nullifying its efficiency advantage.

In adversarial underwater environments, the threats of EOAPs can be summarized as follows:

(1) Natural camouflage. EOAPs can be visually concealed within low-contrast underwater textures such as coral patterns or sand ripples. These perturbations are difficult to distinguish through human vision or simple preprocessing, making them stealthy and dangerous.
(2) Practical constraints. Underwater missions are often extremely sensitive to inference latency. If EOAPs induce doubled or prolonged inference times, tasks may fail or incur severe consequences. For instance, in military scenarios, a delayed AUV detector may fail to recognize underwater mines or hostile targets in time, posing critical safety risks.

In summary, although A-ViT demonstrates remarkable advantages in energy-aware optimization, its dynamic inference mechanism exposes new vulnerabilities. Attackers can exploit EOAPs to negate efficiency gains and undermine mission reliability. Therefore, a key challenge for underwater deployment lies in balancing energy efficiency with adversarial robustness. This motivates the defense strategies that will be further elaborated in the subsequent section.

## 3. Experiments

### 3.1. Quantitative Analysis of Hardware Design and Material Selection Experiments

A parameterized evaluation was performed to assess the Section 2.1 hardware framework using the established power-model equations and a concrete off-the-shelf component profile. Grounded in these selections, quantitative calculations report energy consumption, endurance, and heat-dissipation requirements under three operating modes—low-power (20%), medium-power (80%), and high-load (100%). This device-anchored analysis provides reproducible numerical evidence of feasibility and informs engineering trade-offs for subsequent integration, while remaining consistent with the theory-driven framework.

Specific device models:

(1) Embedded processor: Raspberry Pi 4B (3–5 W).
(2) Imaging module: Raspberry Pi Camera v2.1 (1.2 W).
(3) Illumination unit: 10 W LED, duty ratio adjustable 20–100%.
(4) Power system: 4S2P 18650 Li-ion battery pack, 14.8 V, 6 Ah (88.8 Wh total).

Based on operational depth, three task modes were defined:

(1) Shallow-water mode: LED duty ratio = 20%.
(2) Medium-depth mode: LED duty ratio = 80%.
(3) Deep-water mode: LED full power operation.

This classification simulates typical underwater application scenarios. Using the power equations, we computed average power, endurance, energy per frame, and required heat dissipation. Results are shown in Table 1.

Based on Table 1, endurance is approximately 8.6 h in the low-power mode, 4.9 h in the medium-power mode, and 2.6 h in the high-load mode, aligning, respectively, with long-duration cruising, routine acquisition, and short inspection tasks. Per-frame energy rises from 0.53 J (low-power) to 0.98 J (medium-power) and 1.77 J (high-load), while the required heat-dissipation area ranges from 17.6 cm^2^ to 58.9 cm^2^; under an allowable temperature rise of ≤15 K, these values are attainable using standard aluminum housings.

The analysis is model-based rather than prototype-based, so some deviation is expected due to component tolerances and operating conditions; however, within reasonable error margins the trends remain unchanged, and the hardware design choices—and their role within the proposed detection framework—are not materially affected.

In addition to power and thermal indices, the physical properties of external housing and optical window materials significantly affect long-term reliability. Table 2 summarizes the transparency, thermal conductivity, density, yield strength, seawater corrosion resistance, and cost of typical candidate materials.

**Material Suitability Analysis**

1. **Metals:**
  (1) Aluminum alloy (6061/5083): High thermal conductivity (120–170 W/m·K) and moderate strength (215–275 MPa) allow effective heat dissipation with relatively low weight, suitable for shallow and medium-depth tasks. However, long-term immersion requires anodizing or protective coatings due to limited corrosion resistance.
  (2) Stainless steel (316L): Offers excellent seawater resistance, especially against pitting corrosion, though high density (8.0 g/cm^3^) limits portability.
  (3) Titanium alloy (Ti-6Al-4V): Superior strength (800–900 MPa) and corrosion resistance make it the only viable choice for >300 m deep-sea missions despite higher cost.
2. **Transparent materials**
  (1) PMMA (Acrylic): High transparency (92–93%) and light weight (1.2 g/cm^3^) make it suitable for shallow (<50 m) low-load tasks. However, low strength (65–75 MPa) and poor durability limit deep-sea use.
  (2) Polycarbonate (PC): Slightly lower transparency (88–90%) but better impact resistance than PMMA; suitable for shallow-to-medium depths (<100 m).
  (3) Tempered glass: Transparency 90–92% with yield strength 140–160 MPa, applicable for medium depths (<300 m), though heavier and fracture-prone.
  (4) Sapphire: Optimal candidate for deep-sea windows (>300 m) due to extreme strength (2000–2500 MPa), excellent corrosion resistance, and high optical stability. Cost, however, restricts large-scale use.

**Optimal Structural Solutions**

Integrating the results of Table 1 and Table 2, two structural strategies are proposed:

(1) Fully transparent solution (PMMA/PC/Sapphire): Simplifies optical design but struggles to meet thermal dissipation requirements under high-load conditions; limited to shallow-water, low-power tasks.
(2) Metallic housing and localized transparent window: Metals provide structural strength and heat dissipation, while the transparent window ensures optical imaging. This hybrid solution flexibly adapts to shallow, medium, and deep-water tasks, achieving balanced performance.

Thus, the optimal material recommendations are:

(1) Shallow-water tasks: Aluminum alloy housing + PMMA/PC window.
(2) Medium-depth tasks: Aluminum alloy or stainless steel housing + tempered glass window.
(3) Deep-water tasks: Titanium alloy housing + sapphire window.

This layered material strategy balances mechanical robustness, thermal management, optical stability, and cost across operating regimes. Although the hardware selection is theory-driven, the calculations are rigorous; within reasonable error bounds, the scheme is considered a practical reference for physical system design.

### 3.2. Experimental Analysis of the Image Processing Module

#### 3.2.1. Image Super-Resolution

**Figure 8:**
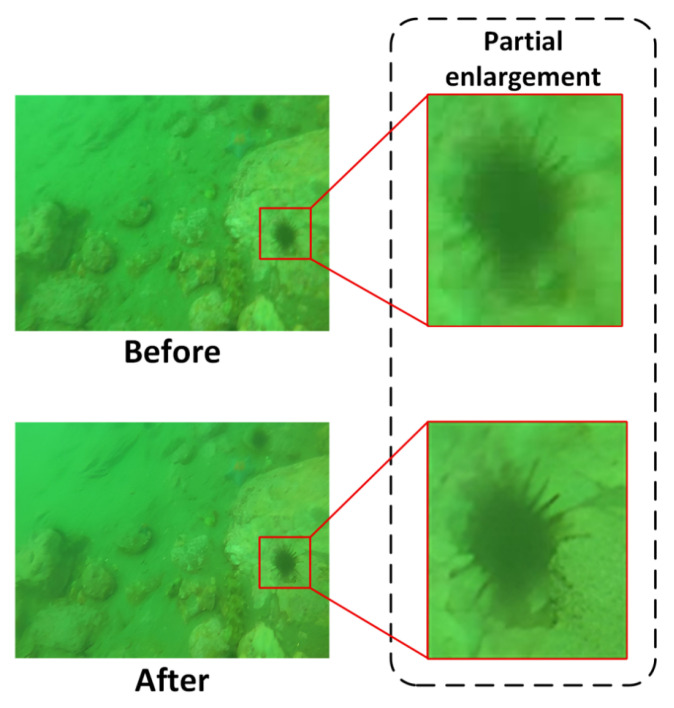
Comparison of underwater image super-resolution results based on HAT.

Figure 8 illustrates the visual comparison of underwater images before and after super-resolution reconstruction. In the blurred image (Before), object boundaries, such as those of marine organisms, are severely smeared, and spiny structures are nearly indistinguishable from the surrounding background. After reconstruction (After), the object boundaries appear sharper, fine details are effectively restored, and the overall perceptual clarity and sharpness of the image are significantly improved.

To further verify the reconstruction effectiveness, Table 3 clearly shows the advantage of HAT in both visual and objective evaluation metrics. Specifically, the PSNR increased from 15.62 dB in blurred images to 27.29 dB after reconstruction, representing a relative improvement of 74.8%, which demonstrates effective suppression of pixel-level noise. The SSIM improved dramatically from 0.186 to 0.885, corresponding to a 375.8% increase, indicating strong recovery of structural similarity and global consistency. Meanwhile, the MSE decreased from 0.0151 to 0.00187, a reduction of 87.6%, confirming enhanced fidelity in pixel-wise reconstruction accuracy.

The results suggest that the HAT-based super-resolution module not only achieves noticeable improvements in visual clarity but also yields consistent advantages in objective metrics. By restoring high-frequency structural details and suppressing noise, the reconstructed images provide more discriminative inputs, which are particularly beneficial for detecting small objects and weak-texture targets in underwater environments.

#### 3.2.2. Multi-Stage Underwater Image Enhancement

**Figure 9:**
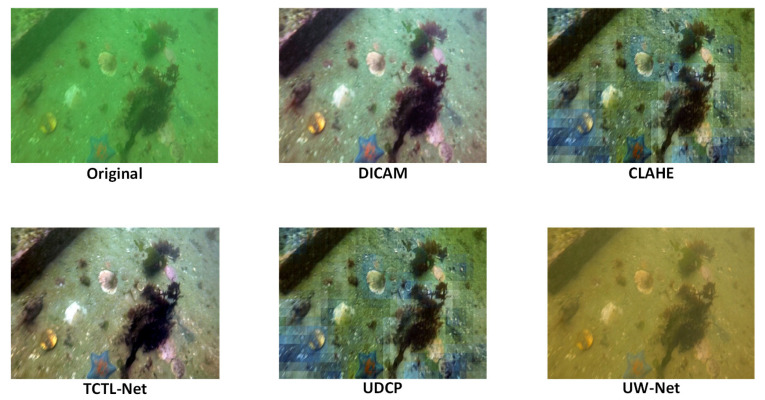
Visual comparison of different underwater image enhancement methods.

Figure 9 illustrates the visual enhancement results of different representative methods for underwater images. The original image exhibits severe color distortion, reduced contrast, and blurred target boundaries. To ensure a scientifically rigorous analysis, the combination of qualitative visual results and quantitative performance metrics from Table 4 will help clarify the strengths and weaknesses of the different methods.

(1) CLAHE (Contrast-limited adaptive histogram equalization):

Visual Analysis: While CLAHE improves local contrast, it amplifies noise, especially in low-texture areas, resulting in visible artifacts. The edges are enhanced but still appear blurry in some areas.

Quantitative Performance: The method shows an improvement in both UIQM (3.42) and UCIQE (0.612), but the MSE_UIQM value (0.179) suggests some loss of texture consistency, confirming the presence of artifacts and unnatural effects.

(2) UDCP (Underwater Dehazing and Color Processing):

Visual Analysis: UDCP does a better job of color restoration but introduces a bluish tint due to over-correction. The edges remain unclear, and texture restoration is incomplete.

Quantitative Performance: UDCP achieves an acceptable UIQM (3.15) and UCIQE (0.587), but with an extremely low MSE_UIQM (0.022), which reflects a partial restoration of textures. However, the lack of consistency in local features (MSE_UCIQE = 0.0016) indicates room for improvement in overall visual quality.

(3) UW-Net (Underwater Image Enhancement Network):

Visual Analysis: UW-Net improves adaptability to complex lighting conditions but introduces noticeable over-correction, especially in green hues, leading to unnatural visual effects. The texture details remain inconsistent, and edge clarity is still not optimal.

Quantitative Performance: UW-Net achieves a UIQM of 3.56 and UCIQE of 0.641, demonstrating a balanced performance. However, the MSE_UIQM (0.313) shows significant deviations in local consistency, confirming the over-correction observed visually.

(4) TCTL-Net (Template-free Color Transfer Learning):

Visual Analysis: TCTL-Net enhances both color and contrast, but at the expense of image sharpness, introducing artifacts and edge distortion. This method amplifies the target contrast but results in unnatural, over-sharpened visuals, especially along the edges.

Quantitative Performance: With a UIQM of 3.71 and UCIQE of 0.665, TCTL-Net performs well in terms of contrast and color enhancement. However, the high MSE_UIQM value (0.500) confirms that the method suffers from over-enhancement and edge distortions, which undermines its visual quality.

(5) DICAM (Deep Inception and Channel-wise Attention Modules):

Visual Analysis: DICAM achieves a balanced enhancement, suppressing color distortions and enhancing edge clarity while maintaining local texture integrity. The method restores a natural look, with a minimal increase in artifacts compared to traditional methods.

Quantitative Performance: DICAM outperforms all other methods, achieving the highest UIQM (3.85) and UCIQE (0.673), reflecting its superior color fidelity and perceptual quality. Although its MSE_UIQM (0.722) and MSE_UCIQE (0.0092) are slightly higher due to stronger pixel-level adjustments, the method achieves an optimal trade-off, offering natural visual realism while ensuring texture consistency.

This section verifies the superiority of the DICAM enhancement adopted in this system through multi-method comparisons, using both visual evidence and quantitative metrics.

### 3.3. Visualization Analysis of YOLOv11-CA_HSFPN Detector

**Figure 10:**
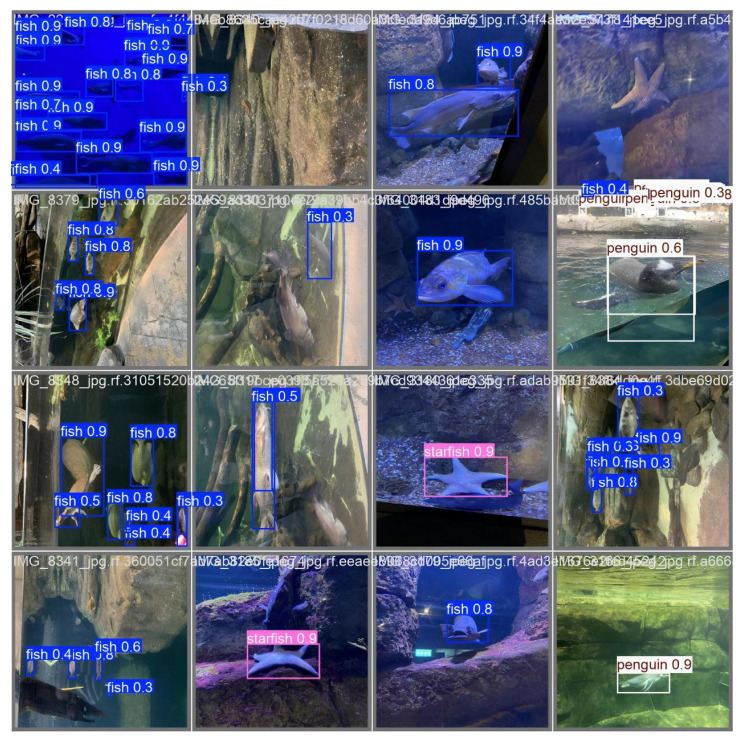
Visualization results of the improved YOLOv11-CA_HSFPN in underwater object detection tasks.

Figure 10 presents the visualization results of the improved YOLOv11-CA_HSFPN model in underwater object detection tasks. Overall, the model maintains stable detection performance under challenging conditions such as complex backgrounds, color distortions, and contrast degradation in underwater imagery. The model can accurately recognize multiple categories of underwater objects including fish, penguins, and starfish. The predicted bounding boxes align well with object contours, with confidence scores typically ranging from 0.7 to 0.9, demonstrating strong capability in both classification and localization. Particularly in densely populated fish scenarios, the model effectively distinguishes adjacent individuals while minimizing false positives and missed detections. This highlights the contribution of the introduced HSFPN module in enhancing multi-scale feature fusion, enabling robust detection across different object scales and complex scenes.

In terms of small object detection, YOLOv11-CA_HSFPN shows significant improvements. Even under long-range observation or partial occlusion, the model can still detect small fish or edge-region targets with high confidence. This can be attributed to the integration of the Coordinate Attention (CA) mechanism, which strengthens target-related feature representation by emphasizing informative channels while suppressing redundant background noise. Compared with the baseline YOLO detector, the improved architecture demonstrates stronger adaptability to underwater optical degradations such as color shifts, low contrast, and scattering blur, thereby achieving superior performance in recognizing small and weak-textured objects.

**Figure 11:**
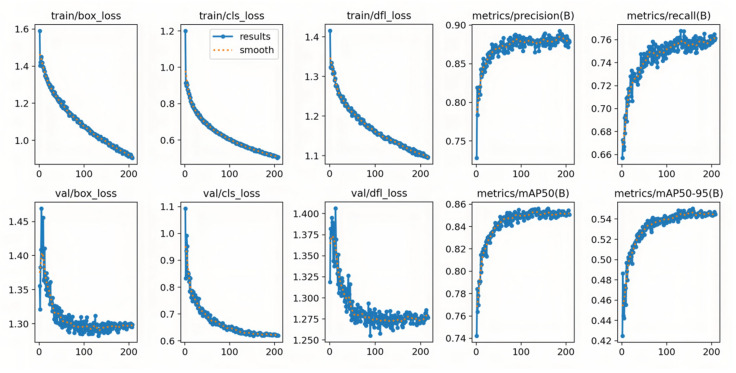
Training and validation convergence curves of the improved YOLOv11-CA_HSFPN.

The visualization results confirm strong adaptability in real-world scenarios, while the convergence characteristics further validate the effectiveness of the structural modifications. As shown in Figure 11, both training and validation losses (box, cls, and dfl) decrease monotonically and gradually converge with increasing iterations. Meanwhile, Precision, Recall, mAP@0.5, and mAP@0.5:0.95 steadily increase and stabilize, reflecting reliable optimization behavior.

The curves exhibit the following properties:

1. Stable Convergence: Validation box_loss and dfl_loss curves show minimal oscillations, indicating smooth convergence.
2. Synchronized Quality Improvement: Precision and Recall consistently improve, and the final mAP plateau suggests strong generalization ability.
3. Effectiveness of Structural Modifications: Compared with the baseline, the CA-HSFPN-enhanced model achieves higher plateaus across metrics, confirming stronger feature representation capacity.

To ensure reproducibility, the training configuration of the improved YOLOv11-CA_HSFPN is summarized in Table 5. The configuration balances memory constraints with training speed and accuracy, following mainstream object detection practices.

### 3.4. Visualization Analysis of Fish-School Detection Based on A-ViT

In underwater fish-school detection tasks, relying solely on numerical indicators such as mAP or Precision/Recall can reflect overall performance but often fails to reveal detailed behavior in complex scenarios. Particularly in underwater environments with densely distributed targets, uneven illumination, and significant background disturbances, intuitive visualization provides a clearer demonstration of differences in spatial localization, confidence stability, and robustness across models. Therefore, this study evaluates detection performance on real fish-school images using both unaltered raw inputs and A-ViT dynamically cropped inputs, aiming to visually validate the trade-off between efficiency and robustness introduced by the mechanism.

**Figure 12:**
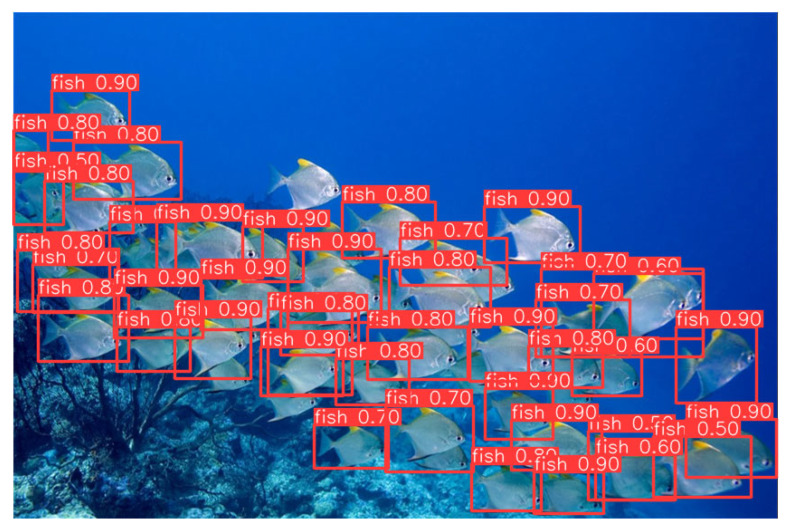
Detection results of YOLOv11-CA_HSFPN on raw input images.

**Figure 13:**
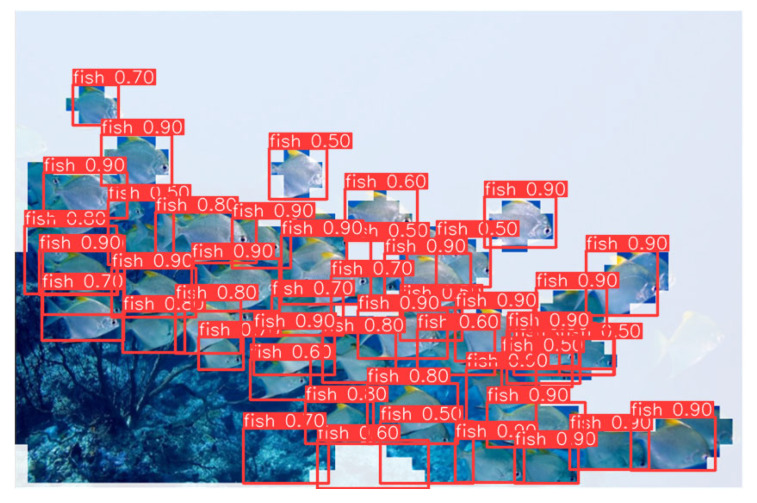
Detection results of YOLOv11-CA_HSFPN on A-ViT cropped input images.

As shown in the comparison analysis of Figure 12 and Figure 13, when dynamic cropping by A-ViT is applied, most fish targets are still correctly identified. However, the detection results exhibit redundancy: duplicate bounding boxes appear around the edges of fish, overlaps among boxes increase, and confidence distributions fluctuate more widely. This phenomenon suggests that while A-ViT’s token pruning reduces redundant computation, it inevitably disrupts spatial continuity, leading to the introduction of more redundant annotations at the detection output level.

It is noteworthy that this redundancy is not purely negative. First, in adversarial settings, perturbations often target background or boundary regions, while A-ViT’s dynamic cropping prioritizes the retention of target-relevant tokens. This ensures comprehensive target coverage, preventing critical missed detections. Consequently, even with redundant bounding boxes, the system maintains effective detection integrity. Second, in safety-critical applications such as autonomous underwater vehicles (AUVs) or long-range monitoring platforms, avoiding missed detections is often more important than reducing redundancy, as false negatives may directly cause mission failure or safety risks.

From a design perspective, the dynamic cropping of A-ViT complements the structural optimization of YOLOv11-CA_HSFPN. The former substantially reduces background computation, enhancing energy efficiency and inference speed, while the latter—through CA and HSFPN modules—improves discrimination of small and densely packed targets. This synergy enables the system to maintain high confidence despite the spatial discontinuities introduced by cropping. Future integration with advanced non-maximum suppression (NMS) strategies or temporal consistency mechanisms may further reduce redundancy, achieving a balanced trade-off among accuracy, efficiency, and adversarial robustness.

In conclusion, the experimental results highlight the dual role of A-ViT in underwater fish-school detection: on the one hand, its dynamic inference significantly lowers computational overhead and boosts energy efficiency; on the other hand, even with increased redundancy, the system retains complete target coverage and high robustness. These characteristics provide unique advantages for deployment in safety-critical underwater applications, demonstrating that A-ViT serves not only as an effective tool for efficiency optimization but also as a promising pathway to enhance adversarial robustness.

### 3.5. Dynamic Visualization Analysis and Adversarial Vulnerability Verification of A-ViT in Underwater Fish-School Detection

To further validate the effectiveness of the A-ViT dynamic inference mechanism proposed in Section 2.4 under complex underwater environments, we conducted visualization experiments on real fish-school images.

**Figure 14:**
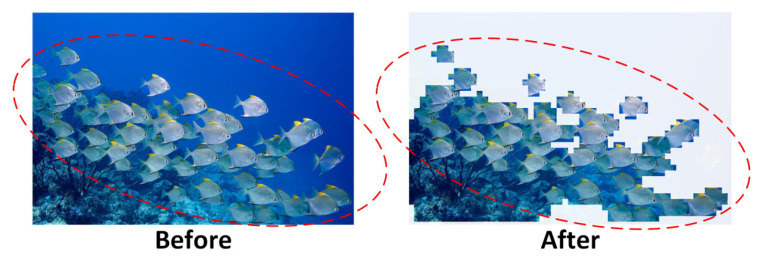
Dynamic token selection results of A-ViT in underwater fish-school detection.

As shown in Figure 14, the left panel presents the raw input, where both fish schools and the surrounding background are fully fed into the network, whereas the right panel illustrates the A-ViT dynamic selection results, where the model focuses on fish-related tokens while cropping or downscaling large background regions. This visualization aligns with theoretical analysis Importantly, the preserved regions are concentrated on fish contours and texture details, consistent with the robustness index I(x), which favors tokens that remain stable under perturbations. This mechanism not only enhances attention to critical regions but also naturally weakens perturbation-prone background areas, thereby improving overall robustness.

From a system perspective, this adaptive property offers two key advantages:

1. Energy efficiency improvement—By reducing redundant background computation, A-ViT compresses the effective processing area to less than half, synchronously decreasing memory consumption and inference latency, thereby significantly improving deployability on embedded platforms.
2. Robustness enhancement—The discarded regions often coincide with locations where adversarial patches can be most effectively hidden, making the dynamic pruning mechanism inherently resistant to such perturbations while lowering energy costs.

In summary, these visualization results empirically confirm the theoretical advantages of A-ViT: its dynamic token selection mechanism not only reduces computational overhead for efficiency but also enhances discriminative robustness in adversarial underwater environments. This finding provides strong empirical support for the dual optimization of energy efficiency and robustness discussed in subsequent sections.

To further examine the impact of adversarial threats, we simulated attacks to evaluate the resilience of the proposed framework under potential hazards. To this end, we adopted the SlowFormer attack as a representative scenario and conducted experiments on fish-school images to reveal its real-world risks in underwater contexts.

**Figure 15:**
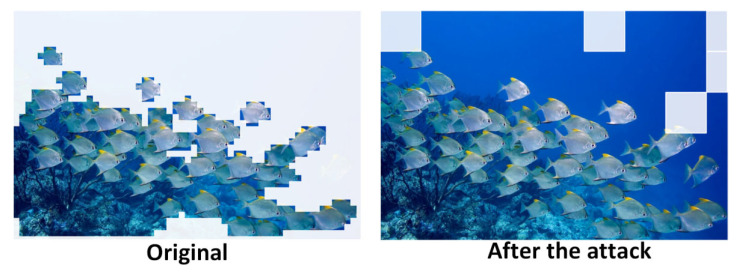
Visualization of A-ViT under SlowFormer attack in underwater fish-school detection.

As shown in Figure 15, after the attack, A-ViT’s token pruning mechanism is heavily disrupted. Normally, the model selectively focuses on fish-related regions while cropping large portions of the background for efficient inference. However, under the SlowFormer attack, the model is forced to retain redundant tokens across multiple areas, even within homogeneous water regions, leading to unnecessary computation. This indicates that the adversarial patch perturbs the halting distribution, delaying token exits and effectively reverting the model to a full ViT inference state.

Critically, this attack not only eliminates A-ViT’s efficiency advantage but also poses severe threats to system energy consumption and robustness. In our discussion, the pruning rate dropped significantly, computation surged, and the device’s power consumption increased nonlinearly, directly reducing endurance on resource-constrained underwater platforms. Meanwhile, excessive attention to background regions reduced the model’s discriminative capacity on fish targets, destabilizing confidence scores. In adversarial scenarios such as military underwater operations, such attacks could increase computational load, impair real-time response, and ultimately weaken mission performance.

These findings reveal the latent risks of energy-oriented adversarial attacks in underwater detection: attackers can undermine deployability without directly reducing detection accuracy, simply by manipulating the computational distribution of the model. This underscores not only the necessity of incorporating adversarial defenses into underwater intelligent perception frameworks but also the importance of achieving a deeper balance between efficiency optimization and adversarial robustness in future detection systems.

### 3.6. Defense Strategy: Image-Stage Quick Defense via IAQ

In the preceding experiments, we demonstrated that under normal conditions, A-ViT can effectively reduce computational complexity through ROI cropping, achieving low-latency and memory-efficient inference. However, when subjected to energy-oriented adversarial attacks such as SlowFormer, the ROI localization mechanism is severely disrupted. This results in abnormal expansion of the input regions forwarded to A-ViT, causing “explosions” in both inference latency and memory usage. To address this vulnerability, we designed a lightweight Image-stage Attack QuickCheck (IAQ) module, serving as a preventive defense mechanism prior to A-ViT inference.

The core principle of IAQ is to fuse low-cost statistical indicators—frequency-domain features, high-frequency energy ratio, local entropy variance, Laplacian sharpness anomaly, color channel shift, and consistency under slight perturbations—into an attack score. If this score exceeds a threshold, the input frame is flagged as a suspicious adversarial sample and bypasses A-ViT, being redirected to the baseline full-image detector. This prevents ROI failure from propagating into A-ViT and fundamentally eliminates latency and memory explosions. The design and execution flow of the defense are illustrated in Appendix A.

Experimental results (Table 6) confirm that IAQ substantially mitigates latency escalation under adversarial conditions. For instance, YOLOv8’s latency under attack is reduced from 13.0 ms to 11.0 ms, while RT-DETR-R50 decreases from 16.8 ms to 11.3 ms. Although IAQ introduces a modest overhead of about +2 ms compared to clean A-ViT+ROI inference, it effectively eliminates latency explosions, keeping system delay within a predictable and bounded range. In other words, IAQ transforms the system’s latency behavior from “highly variable and attack-sensitive” to “bounded and predictable,” providing crucial safeguards for real-time or embedded applications.

From a system perspective, the advantage of IAQ lies not only in avoiding worst-case performance collapse but also in enhancing overall robustness and deployability. Although IAQ adds approximately 1–3 ms latency overhead in benign conditions, it reduces latency under attack by 33–40%, yielding a favorable trade-off. This bounded stability is particularly critical for real-world tasks, where the Worst-Case Execution Time (WCET) often determines whether real-time requirements can be met. Hence, IAQ provides a practical and effective solution for the secure deployment of A-ViT frameworks in adversarial underwater environments.

## 4. Discussion

### 4.1. Ablation Study of Image Processing Modules

Table 7 summarizes the ablation on the two image-processing modules—Color Restoration (CR) and Super-Resolution (SR)—evaluated under the original YOLO detector and our modified YOLO (YOLOv11-CA_HSFPN). Relative to the raw baseline (mAP@0.5 = 54.7; Precision = 0.875; Recall = 0.742; 11.2 M params; 28.6 G FLOPs), introducing CR or SR alone yields consistent gains (A: 55.3/0.880/0.747 with 11.4 M/30.1 G; B: 55.5/0.882/0.750 with 12.0 M/32.7 G). Combining CR+SR provides the strongest improvement within the original detector (C: mAP@0.5 = 56.0; Precision = 0.886; Recall = 0.752) at a still moderate cost (12.3 M; 33.4 G). Replacing the backbone with the modified detector increases accuracy even without preprocessing (D: 56.2/0.889/0.754; 13.1 M; 33.8 G), and the best overall result is obtained when CR+SR is paired with the modified detector (E: mAP@0.5 = 57.0; Precision = 0.892; Recall = 0.760; 13.3 M; 34.5 G). These results support the complementary roles of the two modules in underwater conditions: CR compensates spectral and contrast distortions that confound global color statistics, while SR restores high-frequency structure critical for small or texture-poor targets; the modified detector further capitalizes on the higher-quality inputs to improve localization and confidence calibration.

From an efficiency standpoint, the added computational burden is modest and controlled. Moving from the baseline to the strongest variant (E) increases parameters by only +2.1 M and FLOPs by +5.9 G, while delivering +2.3 mAP@0.5 and higher precision/recall. Among single modules, CR (A) offers the most favorable gain-per-cost profile, delivering +0.6 mAP with the smallest overhead (+0.2 M params; +1.5 G FLOPs), whereas SR (B) provides a slightly larger gain (+0.8 mAP) at higher compute (+0.8 M; +4.1 G). The joint CR+SR configuration (C) achieves the best single-detector accuracy increase (+1.3 mAP) with restrained overhead (+1.1 M; +4.8 G). When the deployment budget is tight, A is a strong choice due to its minimal cost; when a balance between accuracy and efficiency is needed on the original detector, C is preferable; when accuracy is paramount and limited additional compute is acceptable, E is the recommended setting.

Overall, the extended ablation confirms three conclusions aligned with our design goals: (i) CR and SR are synergistic, with CR improving global photometric fidelity and SR enhancing local detail; (ii) architectural optimization of the detector amplifies the benefits brought by improved inputs; and (iii) the accuracy increments introduced by preprocessing and detector refinement are achieved with small, predictable increases in Params and FLOPs, preserving practical deployability for resource-constrained underwater platforms.

### 4.2. Performance Analysis and Ablation Study of YOLOv11-CA_HSFPN

As summarized in Table 8, the proposed YOLOv11-CA_HSFPN advances the accuracy–efficiency frontier for underwater detection at comparable latency to YOLO-family models. It attains the highest accuracy among all candidates—Precision/Recall/mAP@0.5 of 0.889/0.754/56.2%—with a latency of 10.5 ms. Relative to YOLOv11 (54.7% at 10.2 ms), it provides +1.5 mAP for a +0.3 ms latency change. Compared with RT-DETR v2-R50 (55.9% at 12.6 ms) and v1-R50 (55.0% at 14.8 ms), it delivers higher accuracy with lower latency (−2.1 ms and −4.3 ms, respectively), thus achieving a Pareto-superior balance between accuracy and inference time. These results indicate that the proposed high-order feature fusion and coordinate attention in HSFPN convert nearly all added computation into useful representational capacity rather than overhead, improving both localization and confidence calibration in the presence of optical degradation and small-object targets.

Beyond point estimates, the comparative trends in Table 9 clarify the role of architecture in practical deployment. The YOLO lineage (v5→v11) shows steady but limited gains in mAP@0.5 (<2.5 percentage points), suggesting saturation of purely convolutional hierarchies under underwater conditions. Transformer-based RT-DETR models improve global modeling and multi-object interaction, reflected by higher recall and mAP, but incur noticeably longer inference times. YOLOv11-CA_HSFPN closes this gap by retaining YOLO-like throughput while surpassing both YOLO and RT-DETR in accuracy, thereby shifting the empirical accuracy–latency trade-off curve outward. Using latency as a deployment-level proxy for energy usage, the detector reaches a more favorable operating point: for each processed frame it achieves higher detection quality at equal or lower time-per-frame than competing high-accuracy baselines, supporting battery-conscious and thermally constrained underwater platforms.

From an application standpoint, these properties translate into actionable guidance. When strict real-time response is required (e.g., online ROV inspection or rapid subsea pipeline screening), YOLO-family detectors remain attractive due to their short latency; among them, YOLOv11-CA_HSFPN offers the best accuracy without sacrificing speed. For missions that previously favored Transformer detectors for accuracy, the proposed model provides a practical alternative that sustains or improves detection quality while reducing inference time, thereby lowering energy draw over long-duration deployments. Collectively, the evidence demonstrates that targeted architectural refinement—rather than wholesale paradigm shifts—can deliver a superior accuracy–efficiency balance for underwater object detection, aligning with the core objective of enabling robust, real-time operation on resource-limited platforms.

The ablation results in Table 9 further disentangle the contributions of the individual modules. Starting from the baseline (Precision/Recall/mAP@0.5 = 0.875/0.742/54.7; 10.2 ms), adding HSFPN increases mAP@0.5 to 55.6 with a marginal latency change to 10.4 ms. The precision-oriented gain (0.875→0.881) and a modest recall rise (0.742→0.746) indicate that high-order feature fusion mainly strengthens discriminability and localization sharpness, consistent with better feature aggregation across scales. Adding CA alone raises mAP@0.5 to 55.8 at 10.3 ms and notably improves recall (0.742→0.749) with near-baseline precision (0.877), suggesting that coordinate attention predominantly enhances saliency and target recoverability under color cast and low-contrast conditions typical of underwater imagery. Importantly, both modules improve ΔmAP@0.5:0.95 (+0.9 for HSFPN; +1.1 for CA), reflecting accuracy gains that persist at higher IoU thresholds; this indicates better box quality and calibration, not merely looser detections.

When HSFPN and CA are combined (CA_HSFPN), the effects are additive: mAP@0.5 reaches 56.2% with Precision/Recall of 0.889/0.754 at 10.5 ms, and ΔmAP@0.5:0.95 increases to +1.5, the largest among all variants. The joint improvement in both precision and recall implies complementary roles—HSFPN contributes sharper fine-scale representations, while CA stabilizes feature responses against illumination and spectral distortions—leading to higher confidence on true positives and fewer missed small targets. Crucially, these gains are achieved with only +0.3 ms over baseline latency, preserving real-time throughput and, by proxy, energy efficiency per frame. Taken together, the ablation confirms that the proposed design choices are cost-effective: each module independently improves performance at near-constant runtime, and their combination achieves the strongest accuracy with negligible latency overhead, thereby reinforcing the paper’s central claim of attaining a superior balance between detection quality and deployment efficiency for underwater platforms.

### 4.3. Comparative Study with Representative Underwater Detectors

To contextualize the proposed framework within the current landscape, we conducted a head-to-head comparison against representative underwater detection pipelines that couple heterogeneous front-end enhancements with different back-end detectors, including U-DECN (denoising with deformable convolution and a DETR backbone), SWIPENET (multi-scale attention with YOLOv5-S), PE-Transformer (transformer-based enhancement with ViT-DETR), AGS-YOLO (attention with global feature fusion and YOLOv7-Tiny), and NAS-DETR (DETR augmented by neural architecture search).

The consolidated results (see Table 10) exhibit a clear accuracy–efficiency trade-off. PE-Transformer delivers the highest mAP@0.5 (57.3%) with strong precision/recall (0.895/0.762), but it also incurs the largest computational footprint (59.8 M parameters and 78.1 G FLOPs). By contrast, our pipeline—combining HAT-based super-resolution and DICAM-based enhancement, followed by YOLOv11-CA_HSFPN—achieves near-top accuracy (mAP@0.5 = 57.0%, precision = 0.892, recall = 0.760) while using only 13.3 M parameters and 34.5 G FLOPs, corresponding to roughly one quarter to one half of the computational cost of strong transformer-centric baselines. Relative to attention-enhanced YOLO families, our method also offers a more favorable Pareto point: it surpasses AGS-YOLO (56.5%, 14.2 M, 36.0 G) and SWIPENET (55.8%, 15.6 M, 35.9 G) in accuracy without increasing complexity. Compared with DETR-style pipelines, it matches or exceeds U-DECN (56.1%, 37.5 M, 61.2 G) and NAS-DETR (56.8%, 40.4 M, 70.3 G) while requiring substantially fewer resources (see Table 10).

The observed gains arise from two complementary design choices. First, the physics-aware enhancement stage—HAT for super-resolution together with DICAM for color and contrast restoration—recovers high-frequency details and improves the visibility of small or low-contrast targets that are common in underwater imagery. Second, on the detection side, the CA-HSFPN neck integrates coordinate attention with high-order cross-scale feature fusion, which stabilizes recall and tightens localization while adding only negligible latency. Together, these choices shift the accuracy–efficiency frontier outward: the system approaches the detection quality of PE-Transformer at a fraction of the computational budget and outperforms attention-augmented YOLO baselines in both mAP and resource usage. From a deployment perspective, lower parameter counts and FLOPs translate into reduced latency and energy per frame, which is essential for autonomous underwater vehicles, remotely operated vehicles, and other embedded platforms. Overall, the cross-model comparison substantiates the central claim that a co-designed, physically grounded enhancement stage paired with a lightweight, attention-aware detector provides a practical, Pareto-efficient solution for real-time underwater perception (see Table 10).

### 4.4. Experimental Analysis of the A-ViT+ROI Dynamic Inference Mechanism

To evaluate the energy–efficiency performance of A-ViT+ROI dynamic inference under varying foreground ratios (FR), we conducted systematic experiments on three representative detectors: YOLOv8, YOLOv11-CA_HSFPN, and RT-DETR-R50 (see Table 11).

Overall, A-ViT demonstrates significant reductions in inference latency and memory usage under low-to-moderate foreground ratios (FR ≈ 0.2–0.4). For example, with YOLOv8 at FR = 0.23, the latency decreased to 7.18 ms, representing a 34.7% reduction compared to the baseline, while memory usage dropped to 0.55 GB, a reduction of 75.0%. Similar patterns were observed with YOLOv11-CA_HSFPN and RT-DETR-R50, achieving latency reductions of 27.3% and 48.9%, and memory savings of 74.6% and 80.0%, respectively, at FR ≈ 0.24 and 0.18. These results indicate that the dynamic token pruning mechanism of A-ViT is particularly effective in sparse-target scenarios, where redundant computations can be avoided, thereby improving real-time performance and hardware efficiency.

However, as the foreground ratio increases (FR ≈ 0.6), the efficiency advantage of A-ViT diminishes or even reverses. For example, with YOLOv8 at FR = 0.62, latency rises to 12.38 ms, an increase of 12.5% over the baseline, while memory usage grows to 2.31 GB. Similar phenomena are observed for YOLOv11-CA_HSFPN (FR ≈ 0.59) and RT-DETR-R50 (FR ≈ 0.65), with latency increases of 6.6% and 3.2%, respectively, memory consumption increasing by about 5%, and fallback mechanisms triggered (Fallback = TRUE). This suggests that in dense-target scenarios, A-ViT must retain more tokens to avoid missed detections, rendering dynamic pruning ineffective and inference close to full computation.

Differences among detectors further reveal architectural characteristics. RT-DETR-R50 achieves the most substantial efficiency gains under low FR, reducing latency by 48.9% and memory by 80.0% at FR = 0.18, reflecting the strong optimization potential of Transformer-based architectures when combined with A-ViT. However, its efficiency also degrades more sharply as FR increases, highlighting its sensitivity to dense target distributions. By contrast, YOLOv11-CA_HSFPN maintains more balanced stability, still achieving a 19.0% latency reduction and 58.9% memory saving at FR ≈ 0.44, showing greater resilience in medium-density conditions. These differences imply that the effectiveness of A-ViT depends not only on foreground ratios but also on the detector’s feature modeling strategy: convolutional architectures demonstrate stability at moderate densities, while Transformers show stronger gains in sparse scenarios.

It should be noted that the FR values for different detectors are not perfectly aligned: FR = 0.23 for YOLOv8, 0.24 for YOLOv11-CA_HSFPN, and 0.18 for RT-DETR-R50. This discrepancy arises from structural differences that affect saliency partitioning. Variations in receptive fields, attention distributions, and feature sensitivities between convolutional and Transformer architectures naturally lead to slight shifts in foreground estimation. Such differences are expected in dynamic inference across architectures and do not affect the overall observed trend.

From an application perspective, these results indicate that A-ViT significantly enhances energy efficiency in underwater tasks with sparse or moderately dense foregrounds, such as long-range monitoring, underwater pipeline inspection, or sparse marine life recognition. However, in dense-target scenarios, such as fish schools or complex seabed topography, the advantages diminish and may even reverse due to fallback-induced overhead. Future research should therefore focus on enhancing the robustness of dynamic pruning in high-density scenarios—for example, through more stable saliency evaluation, multi-scale dynamic scheduling strategies, or hybrid token-retention mechanisms—to ensure efficiency gains remain consistent across diverse application conditions.

### 4.5. Energy–Efficiency Vulnerability of A-ViT+ROI Under Adversarial Attacks

In adversarial experiments (see Table 12), the energy–efficiency performance of A-ViT+ROI degraded severely, with inference latency and memory consumption increasing across all foreground ratios, and fallback mechanisms being universally triggered. In other words, adversarial perturbations undermine A-ViT’s sparsity adaptation, forcing it to revert to near full-scale computation under all conditions.

More critically, the universal triggering of Fallback = TRUE indicates that attacks not only increase resource consumption but also directly disable A-ViT’s dynamic pruning mechanism. The core strength of A-ViT lies in selectively retaining salient tokens to reduce redundant computation while maintaining accuracy. However, adversarial perturbations shift saliency distributions, misleading the model into marking large background regions as “high-saliency.” Consequently, nearly all tokens are retained, forcing full-image inference. This systemic failure highlights the essence of the attack: not degrading detection accuracy directly, but undermining efficiency by collapsing the pruning logic, thereby inflating energy costs across all scenarios.

Performance differences across detectors further reveal architectural vulnerabilities. For YOLOv8 and YOLOv11-CA_HSFPN, latency and memory usage in low-FR conditions increased by 15–18% and 10–15%, respectively. In contrast, RT-DETR-R50 suffered the most, with latency consistently exceeding 16 ms and memory usage approaching 4 GB, indicating the largest efficiency loss. This suggests that Transformer-based architectures are more susceptible to amplification effects under energy-oriented attacks. Their global modeling characteristics make them prone to large-scale misclassification of background tokens as “critical regions,” magnifying resource overhead. In comparison, YOLO-based models also lost sparsity advantages under attack but exhibited smaller absolute increases in resource usage, suggesting that convolutional structures maintain relatively more stability when dynamic inference is compromised.

Mechanistically, this vulnerability stems from A-ViT’s heavy reliance on saliency estimation. Current A-ViT implementations often depend on attention weights or entropy measures to determine token importance, both of which are highly sensitive to adversarial perturbations. Prior studies confirm similar limitations: DynamicViT [36] exhibits performance drops in dense scenarios, Token Merging [37] suffers from robustness issues in complex distributions, and SlowFormer [38] reveals a fundamental tension between efficiency optimization and adversarial robustness. Collectively, these findings support our results—while dynamic inference excels in sparse conditions, it inevitably degrades or fails under adversarial environments or dense-target scenarios.

From an application perspective, this efficiency vulnerability is particularly dangerous in underwater missions. For long-duration robotic cruises or continuous unmanned platform monitoring, endurance heavily depends on inference efficiency. Once subjected to energy-oriented attacks, systems face not only reduced detection accuracy but also sharp increases in power consumption, shortening mission duration and risking outright task failure. Unlike conventional accuracy-oriented attacks, energy attacks are more insidious, as their primary damage lies in resource exhaustion—posing direct threats to mission sustainability and platform safety.

### 4.6. Failure Case Analysis

The summary of the failure cases is shown in Table 13.

In the image-enhancement stage, perceptual quality improves but artifacts can affect detection. Classical local-contrast operations (e.g., CLAHE) may amplify noise or halos, and learning-based enhancement can over-correct color channels; on weak-texture targets these alterations sometimes coincide with elevated false positives or minor box drift. These effects are visible in the side-by-side comparisons in Figure 9 and align with the quantitative trends in Table 4, where gains in UIQM/UCIQE do not strictly guarantee proportional improvements in detection.

In dense scenes with many small, contiguous targets, ROI-based dynamic cropping (A-ViT+ROI) preserves overall coverage but introduces spatial discontinuities at crop boundaries. This manifests as duplicate or partially overlapping boxes near retained-region edges and greater dispersion of confidence scores, as shown by the crowded-scene overlays in Figure 12 and Figure 13. The pattern reflects a bias toward suppressing false negatives in crowded settings at the cost of redundancy and increased post-processing load, while recall remains comparatively stable.

Across foreground-ratio (FR) sweeps, the efficiency benefits of dynamic inference depend strongly on scene occupancy. When the foreground occupies a substantial portion of the frame (upper-midrange in our data), the effective prune rate declines, latency and peak memory increase, and fallback is more frequently activated. The aggregate behavior approaches full-image computation, as summarized in Table 11 and illustrated mechanistically by the ROI focusing in Figure 14, reducing the accuracy–efficiency advantage observed at low-to-mid FR.

Under energy-oriented adversarial perturbations (SlowFormer/EOAP), the learned halting distribution is perturbed and the model retains a larger token set—even in visually homogeneous water regions—eroding latency/memory gains and shifting toward near non-pruned operation. This efficiency collapse is documented in Table 12 and visualized in Figure 15, indicating externally induced destabilization of early-exit behavior rather than a proportional loss of classification capacity.

Finally, the IAQ gate stabilizes worst-case latency by diverting suspicious inputs to a baseline path, as reflected in the attack-condition comparisons in Table 6. In clean conditions it introduces a modest, predictable preprocessing overhead without altering the qualitative ranking of detector variants. Overall, across the reported experiments, the framework maintains a favorable accuracy–efficiency balance in low- to mid-foreground, non-adversarial settings, with principal boundary cases arising in highly crowded scenes and under targeted perturbations.

## 5. Conclusions

This study investigated an integrated underwater object detection framework that unifies image enhancement, lightweight detection, and adaptive inference within an energy-aware design. The following key findings were obtained:

(1) The study verified that underwater object detection can maintain stable and reliable performance even under severe optical degradation and hardware constraints when energy efficiency and robustness are optimized jointly rather than separately.
(2) The coupling of dynamic token pruning and energy-aware modeling enables a self-adjusting perception process in which computational load automatically adapts to scene complexity, achieving efficiency without degrading detection stability.
(3) Analysis of the A-ViT mechanism revealed that while adaptive inference improves energy utilization, it also introduces potential security risks under adversarial or energy-limited conditions—demonstrating that adaptability itself can become a new attack surface.
(4) These insights highlight that future underwater vision systems must treat energy efficiency, stability, and security as interdependent objectives, not isolated optimization targets. This represents a conceptual shift toward trustworthy and resource-adaptive underwater perception.

Despite these encouraging results, several limitations remain. First, the experiments primarily focused on optical underwater imagery, whereas sonar and multispectral modalities were not yet incorporated, limiting the framework’s generality across heterogeneous sensing systems. Second, while the A-ViT demonstrated strong performance under moderate foreground sparsity, its efficiency advantage decreased in dense-target scenarios due to fallback activation. Third, although the IAQ module successfully contained energy-oriented attacks, its defense remains primarily heuristic and may not generalize against evolving attack strategies or unseen perturbation types.

Future research should therefore focus on three directions:

(1) Multi-modal integration: Extending the framework to fuse optical, sonar, and acoustic imaging will enhance robustness under severe visibility degradation.
(2) Adaptive density perception: Developing density-aware dynamic pruning mechanisms can sustain efficiency gains even in dense-target environments.
(3) Unified security–efficiency optimization: Combining data-driven adversarial training with lightweight defense priors will enable a more systematic and theoretically grounded resilience framework.

In summary, this work demonstrates that efficiency and robustness need not be mutually exclusive in underwater perception. Through coordinated optimization of image enhancement, adaptive modeling, and adversarial defense, the proposed approach establishes a promising foundation for next-generation intelligent and secure underwater detection systems.

## Figures and Tables

**Table 1:** Theoretical quantitative indicators under different task modes.

Mode	$\mathrm{Average}\;\mathrm{Power}\;\bar{P}$ (W)	$\mathrm{Endurance}\;T_{\mathrm{run}\;}$ (h)	$\mathrm{Energy}\;\mathrm{per}\;\mathrm{Frame}\;E_{\mathrm{frame}\;}$ (J/Frame, F = 15 fps)	$\mathrm{Energy}\;\mathrm{per}\;1000\;\mathrm{Frames}\;E_{1000}$ (Wh/1000 f)	Required Heat Dissipation Area A (cm^2^)
Low-power cruise	7.9	8.6	0.53	0.15	17.6
Medium-power acquisition	14.7	4.9	0.98	0.27	32.7
High-load inspection	26.5	2.6	1.77	0.49	58.9

**Table 2:** Physical properties and applicability of housing and window materials.

Material	Transparency (%)	Thermal Conductivity k (W/m·K)	Density ρ (g/cm^3^)	$\mathrm{Yield}\;\mathrm{Strength}\sigma_{y}$ (MPa)	Corrosion Resistance in Seawater	Cost
Aluminum alloy (6061/5083)	Opaque	120–170	2.7	215–275	Medium (requires anodizing)	Low
Stainless steel (316L)	Opaque	15–17	8.0	170–290	High (excellent pitting resistance)	Medium
Titanium alloy (Ti-6Al-4V)	Opaque	6–7	4.4	800–900	Excellent	High
PMMA (Acrylic)	92–93	0.18–0.22	1.2	65–75	Poor	Low
PC (Polycarbonate)	88–90	0.18–0.22	1.2	70–85	Poor–Medium	Low–Medium
Tempered glass	90–92	0.9–1.1	2.5	140–160	Medium	Medium
Sapphire	>90	30–40	3.9	2000–2500	Excellent	High

**Table 3:** Comparison of image quality evaluation metrics before and after underwater image super-resolution.

Indicator Name	Blurred Image Value	Super-Resolved Image Value	Improvement
PSNR ↑ (dB)	15.62 dB	27.29 dB	+74.8%
SSIM ↑	0.186	0.885	+375.8%
MSE ↓	0.0151	0.00187	−87.6%

Note: ↑ indicates that higher values correspond to better performance, while ↓ indicates that lower values correspond to better performance. PSNR: Measures fidelity to the reference. SSIM: Measures perceived structural/contrast/luminance similarity to the reference. MSE: Measures pixel-wise error relative to the reference.

**Table 4:** Quantitative performance evaluation of multi-stage underwater image enhancement methods.

Method	UIQM ↑	UCIQE ↑	MSE_UIQM ↓	MSE_UCIQE ↓
Original	3.00	0.550	–	–
CLAHE	3.42	0.612	0.179	0.0038
UDCP	3.15	0.587	0.022	0.0016
UW-Net	3.56	0.641	0.313	0.0051
TCTL-Net	3.71	0.665	0.500	0.0078
DICAM	3.85	0.673	0.722	0.0092

Note: ↑ indicates that higher values correspond to better performance, while ↓ indicates that lower values correspond to better performance. UIQM: Underwater Image Quality Measure—a perceptual index tailored for underwater imagery that combines colorfulness, sharpness, and contrast. UCIQE: Underwater Color Image Quality Evaluation—a quality index derived from chroma variance, mean saturation, and luminance contrast. MSE_UIQM: Mean squared error of the UIQM score between the enhanced result and the reference, reflecting deviation and stability. MSE_UCIQE: Mean squared error of the UCIQE score between the enhanced result and the reference.

**Table 5:** Training configuration of the improved YOLOv11-CA_HSFPN.

Project	Parameter Value
GPU model	NVIDIA RTX 4090 24 GB
Learning Rate	1 × 10^−3^ (cosine decay)
Batch Size	32
Input image resolution	640 × 640
Optimizer	AdamW (β_1_ = 0.9, β_2_ = 0.999)
Weight decay	5 × 10^−4^
Warmup epochs	3
Total training epochs	300
Model checkpoint interval	Every 10 epochs

**Table 6:** Inference latency (ms) under clean and adversarial conditions with and without IAQ defense.

Model	Clean (No Attack)	Attack (No Defense)	Attack (IAQ Defense)
YOLOv8	7.7 ms	13.0 ms	11.0 ms
YOLOv11-C	8.5 ms	12.2 ms	11.1 ms
RT-DEIR-R50	9.9 ms	16.8 ms	11.3 ms

**Table 7:** Ablation study results of image processing modules.

Variant	Image Processing	Detector	mAP@0.5 ↑	Precision ↑	Recall ↑	Params (M) ↓	FLOPs (G) ↓
Baseline	None (Raw)	Original YOLO	54.7	0.875	0.742	11.2	28.6
A	CR	Original YOLO	55.3	0.880	0.747	11.4	30.1
B	SR	Original YOLO	55.5	0.882	0.750	12.0	32.7
C	CR + SR	Original YOLO	56.0	0.886	0.752	12.3	33.4
D	None	Modified YOLO	56.2	0.889	0.754	13.1	33.8
E	CR + SR	Modified YOLO	57.0	0.892	0.760	13.3	34.5

Notes: CR (DICAM) = Color Restoration; SR (HAT) = Super Resolution; Original YOLO = YOLOv11; Modified YOLO = YOLOv11-CA_HSFPN. Note: ↑ indicates that higher values correspond to better performance, while ↓ indicates that lower values correspond to better performance.

**Table 8:** Performance comparison of different detectors on the underwater target detection task.

Variant	Precision ↑	Recall ↑	mAP@0.5 ↑	Latency (ms) ↓
YOLOv5	0.861	0.728	52.6	12.4
YOLOv7	0.868	0.735	53.9	11.7
YOLOv8	0.873	0.739	54.4	11.0
YOLOv10	0.878	0.741	54.9	10.6
YOLOv11	0.875	0.742	54.7	10.2
RT-DETR v1-R50	0.870	0.741	55.0	14.8
RT-DETR v2-R50	0.878	0.749	55.9	12.6
YOLOv11-CA_HSFPN	0.889	0.754	56.2	10.5

Note: ↑ indicates that higher values correspond to better performance, while ↓ indicates that lower values correspond to better performance.

**Table 9:** Ablation study results of the YOLOv11-CA_HSFPN modules.

Variant	Precision ↑	Recall ↑	mAP@0.5 ↑	ΔmAP@0.5:0.95	Latency (ms) ↓
Baseline	0.875	0.742	54.7	-	10.2
+HSFPN	0.881	0.746	55.6	0.9	10.4
+CA	0.877	0.749	55.8	1.1	10.3
+CA_HSFPN	0.889	0.754	56.2	1.5	10.5

Note: ↑ indicates that higher values correspond to better performance, while ↓ indicates that lower values correspond to better performance.

**Table 10:** Comparative performance and complexity of representative underwater detectors.

Model	Image Processing	Detector	mAP@0.5 ↑	Precision ↑	Recall ↑	Params (M) ↓	FLOPs (G) ↓
U-DECN	Denoising + Deformable Conv	DETR	56.1	0.888	0.751	37.5	61.2
SWIPENET	Multi-scale Attention	YOLOv5-S	55.8	0.883	0.748	15.6	35.9
PE-Transformer	Transformer-based enhancement	ViT-DETR	57.3	0.895	0.762	59.8	78.1
AGS-YOLO	Attention + Global Fusion	YOLOv7-Tiny	56.5	0.888	0.755	14.2	36.0
NAS-DETR	None	DETR + NAS	56.8	0.890	0.757	40.4	70.3
Ours	HAT + DICAM	YOLOv11-CA_HSFPN	57.0	0.892	0.760	13.3	34.5

Note: ↑ indicates that higher values correspond to better performance, while ↓ indicates that lower values correspond to better performance.

**Table 11:** Energy–efficiency performance of A-ViT+ROI under different foreground ratios.

Foreground Ratio	Model	A-ViT+ROI Latency (ms)	Latency Reduction (%)	A-ViT+ROI VRAM (GB)	VRAM Reduction (%)	Fallback
0.23	YOLOv8	7.18	34.7	0.55	75.0	FALSE
0.41	YOLOv8	7.77	29.4	0.92	58.2	FALSE
0.62	YOLOv8	12.38	−12.5	2.31	−5.0	TRUE
0.24	YOLOv11-CA_HSFPN	7.63	27.3	0.71	74.6	FALSE
0.44	YOLOv11-CA_HSFPN	8.51	19.0	1.15	58.9	FALSE
0.59	YOLOv11-CA_HSFPN	11.19	−6.6	2.95	−5.4	TRUE
0.18	RT-DETR-R50	7.55	48.9	0.72	80.0	FALSE
0.43	RT-DETR-R50	9.88	33.2	1.49	58.6	FALSE
0.65	RT-DETR-R50	15.27	−3.2	3.78	−5.0	TRUE

**Table 12:** Energy–efficiency performance of A-ViT+ROI under adversarial conditions.

Foreground Ratio	Model	A-ViT+ROI Latency (ms)	Latency Reduction (%)	A-VIТ+ROI VRAM (GB)	VRAM Reduction (%)	Fallback
0.23	YOLOv8	12.83	−16.6	2.50	−13.6	TRUE
0.41	YOLOv8	12.95	−17.7	2.57	−16.8	TRUE
0.62	YOLOv8	13.12	−19.3	2.61	−18.6	TRUE
0.24	YOLOv11-CA_HSFPN	12.01	−14.4	3.07	−9.6	TRUE
0.44	YOLOv11-CA_HSFPN	12.17	−15.9	3.13	−11.8	TRUE
0.59	YOLOv11-CA_HSFPN	12.36	−17.7	3.21	−14.6	TRUE
0.18	RT-DETR-R50	16.57	−12.0	3.92	−8.9	TRUE
0.43	RT-DETR-R50	16.78	−13.4	3.99	−10.8	TRUE
0.65	RT-DETR-R50	16.96	−14.6	4.06	−12.8	TRUE

**Table 13:** Software-side failure cases.

Case	Core Takeaway	Evidence
Enhancement side effects	Better perceptual quality does not guarantee better detection; artifacts/color shifts can induce false positives	Figure 9; Table 4
ROI-cropping redundancy	In dense scenes, recall is preserved but redundant/overlapping boxes emerge at boundaries	Figure 12 and Figure 13
Foreground-ratio dependence	At high foreground ratio, pruning benefits vanish and computation approaches full-image	Table 11; Figure 14
Adversarial efficiency collapse	Adversarial perturbations break early-exit, eliminating efficiency advantages	Table 12; Figure 15
IAQ stabilization	Under attack, IAQ stabilizes worst-case latency; overhead is acceptable in clean runs	Table 6

## Data Availability

The experimental data format of this article is not available for public disclosure.

## Abbreviations

**Abbreviation**	**Full English Name**
A-ViT	Adaptive Vision Transformer.
ViT	Vision Transformer.
HAT	Hybrid Attention Transformer (for Image Restoration).
DICAM	Deep Inception and Channel-wise Attention Modules.
CA	Coordinate Attention.
HSFPN	High-order Spatial Feature Pyramid Network.
YOLO	You Only Look Once (family of one-stage object detectors).
RT-DETR	Real-Time Detection Transformer.
ROI	Region of Interest.
FR	Foreground Ratio.
VRAM	Video Random-Access Memory.
FLOPs	Floating-Point Operations (per second used as compute proxy).
Params	Parameters (model size).
mAP@0.5	mean Average Precision at IoU = 0.5.
ΔmAP@0.5:0.95	Change in mean Average Precision averaged over IoU thresholds 0.50–0.95
IoU	Intersection over Union.
PSNR	Peak Signal-to-Noise Ratio.
SSIM	Structural Similarity Index.
UIQM	Underwater Image Quality Measure.
UCIQE	Underwater Color Image Quality Evaluation.
IAQ	Image-stage Attack QuickCheck.
EOAP	Energy-Oriented Adversarial Patch.
KL (divergence)	Kullback–Leibler Divergence.
WCET	Worst-Case Execution Time.
DCT	Discrete Cosine Transform.
ROV	Remotely Operated Vehicle.
UUV	Unmanned Underwater Vehicle.
CLAHE	Contrast-Limited Adaptive Histogram Equalization.
UDCP	Underwater Dark Channel Prior.
UW-Net	Underwater Net (enhancement network).
TCTL-Net	Template-free Color Transfer Learning Network.
SlowFormer	A representative adversarial attack scenario used in experiments.

## Appendix A. IAQ-Gated Defense Against SlowFormer Attacks

**Algorithm A1. IAQ-Gated Defense against SlowFormer Attacks**
**Input: frame x** **Output: detection result y** **# Step 1: Lightweight feature extraction** **function extract_features(x):** ** R_HF ← DCT_highfreq_ratio(x) # High-frequency energy ratio** ** σ_H^2^ ← local_entropy_variance(x) # Local entropy variance** ** V_Lap ← Laplacian_variance(x) # Sharpness anomaly** ** Δ_c ← color_shift(x) # Color channel shift** ** U ← stability_consistency(x) # Consistency under slight perturbation** ** return [R_HF, σ_H^2^, V_Lap, Δ_c, U]** **# Step 2: Attack score computation** **function IAQ_gate(x):** ** feats = extract_features(x)** ** s ← normalize(feats)** **⋅** **w # z-score + linear weighting** ** if s ≥ τ:** ** return "BYPASS" # Suspicious → bypass A-ViT** ** else:** ** return "A-ViT" # Normal image → use ROI + ViT** **# Step 3: Main inference flow** **for each frame x_t:** ** gate ← IAQ_gate(x_t)** ** if gate == "A-ViT":** ** y ← run_AViT_ROI_detector(x_t)** ** else:** ** y ← run_baseline_detector(x_t)** ** output y**
